# Neutral Models of Microbiome Evolution

**DOI:** 10.1371/journal.pcbi.1004365

**Published:** 2015-07-22

**Authors:** Qinglong Zeng, Jeet Sukumaran, Steven Wu, Allen Rodrigo

**Affiliations:** Biology Department, Duke University, Durham, North Carolina, United States of America; University of Texas at Austin, UNITED STATES

## Abstract

There has been an explosion of research on host-associated microbial communities (i.e.,microbiomes). Much of this research has focused on surveys of microbial diversities across a variety of host species, including humans, with a view to understanding how these microbiomes are distributed across space and time, and how they correlate with host health, disease, phenotype, physiology and ecology. Fewer studies have focused on how these microbiomes may have evolved. In this paper, we develop an agent-based framework to study the dynamics of microbiome evolution. Our framework incorporates neutral models of how hosts acquire their microbiomes, and how the environmental microbial community that is available to the hosts is assembled. Most importantly, our framework also incorporates a Wright-Fisher genealogical model of hosts, so that the dynamics of microbiome evolution is studied on an evolutionary timescale. Our results indicate that the extent of parental contribution to microbial availability from one generation to the next significantly impacts the diversity of microbiomes: the greater the parental contribution, the less diverse the microbiomes. In contrast, even when there is only a very small contribution from a constant environmental pool, microbial communities can remain highly diverse. Finally, we show that our models may be used to construct hypotheses about the types of processes that operate to assemble microbiomes over evolutionary time.

## Author Summary

Microbial communities associated with animals and plants (i.e., microbiomes) are implicated in the day-to-day functioning of their hosts. However, we do not yet know how these host-microbiome associations evolve. In this paper, we develop a computational framework for modelling the evolution of microbiomes. The models we use are neutral, and assume that microbes have no effect on the reproductive success of the hosts. Therefore, the patterns of microbiome diversity that we obtain in our simulations require a minimal set of assumptions relating to how microbes are acquired and how they are assembled in the environment. Despite the simplicity of our models, they help us understand the patterns seen in empirical data, and they allow us to build more complex hypotheses of host-microbe dynamics.

## Introduction

Microbial communities associated with animals and plants (i.e., microbiomes) are implicated in the day-to-day functioning of their hosts in a variety of ways; microbes provide hosts with access to nutrients [1–4], they protect against pathogens [5,6], confer drought resistance [7], mediate hosts’ social interactions [8], and modulate host behavior [9–11]. Not surprisingly, many studies have related human health to the human microbiome. Researchers have proposed that perturbations in human microbiome composition may be associated with a range of disorders, including inflammatory bowel disease [12–14], anxiety and depression [15], obesity [16,17], autism [18,19], allergic responses [20] and respiratory ailments [21–23]. There is a growing number of large-scale projects underway to characterize and analyze the collection of microbes linked to human health and disease using advanced sequencing methods and technologies, including the Human Microbiome Project [24] and the European Metagenomics of the Human Intestinal Tract [25].

Many microbiome studies focus on descriptions of microbial dynamics within hosts over short periods [26–28], or on the composition and diversity of microbial communities amongst hosts with different phenotypes or under different treatment regimes [17,29], but there is increasing recognition that host-microbe dynamics need to be studied within the context of a unified ecological or evolutionary framework. In a recent paper, Costello et al [30] listed four ecological processes that mediate the diversity of human microbiota: environmental selection, whereby the host environment favors the presence and persistence of certain microbial taxa; historical contingency, in which differences in the timing and order of microbial colonization leads to differences in succession and climax communities; random sampling, where stochastic factors influence community assemblages; and dispersal limitation, where the availability of microbial taxa is restricted by the local structure of host communities and environments. Yeoman et al [31] discuss many of these same factors, and frame the challenge of understanding microbial diversity through the lens of evolution, identifying selection induced by competition between individuals of the same or different microbial species, and host influences including phylogeny, as important drivers of host-microbial variation.

In this paper, we take the first steps in developing an explicit framework for modelling the evolutionary and ecological dynamics of microbial communities within a population of hosts. The models we use are related to some of those that metacommunity theorists work with [32,33], specifically, neutral models of ecology and biodiversity [34], but there is one significant difference. Standard spatial models of community assembly, biogeography and biodiversity assume that areas available for colonization remain indefinitely and are static. These models do not apply to the evolution of microbiomes because the “spaces” these microbial communities colonize–that is, the hosts–share an evolutionary history characterized by lineages that persist or go extinct. For this reason, the pairing of host genealogy with microbiome assembly may lead to different patterns of biodiversity than those expected under existing models of microbial community assembly and metacommunity theory.

We develop an agent-based framework, similar to that used recently by Hellweger et al [35], to capture the emergent patterns of microbial diversity over many host generations. In contrast to Hellweger et al, we do not model mutations and speciation in microbial lineages but focus instead on how ecological and evolutionary sampling processes affect the standing variation of microbes in the environment and hosts. The neutral processes that are encapsulated in our framework are based on those that have been proposed by others [30,31] with the added dimensions of an evolutionary timescale and a genealogy of hosts, both of which contribute to shaping the eventual composition and variation of microbial communities within and between hosts. Our models do not assume that hosts differ in their reproductive success as a consequence of their microbial communities; nor do our models assume differences amongst microbes in their propensities to persist in the host or the environment, or in their abilities to disperse. Consequently, whereas our models do not capture the complexity of processes that are likely to mediate the evolution of microbiomes, they serve as minimalist null models against which empirical patterns may be compared.

Our framework is a generic one, and we have not developed it specifically for any one species of host, or indeed, any one systemic compartment within a host. Instead, our models incorporate simple host mechanisms for microbial acquisition, and we explore the effects of parental inheritance of the microbiome, and microbial recruitment from the environment. Our results indicate that parental inheritance tends to reduce microbial diversity and increase homogeneity within hosts, while ongoing environmental acquisition works to maintain microbial heterogeneity within hosts. Interestingly, we observe a non-linear relationship between the degree of parental inheritance and between-host differences in microbiome composition. Finally, we show how these neutral patterns allow us to make predictions about the processes that are important in shaping microbiome diversity when applied to empirical data.

### The models

Our framework applies to a population of hosts and an available pool of microbial colonists. As a first step, we assume that hosts do not exert any preferences on the microbial taxa they acquire. Similarly, we assume that microbes do not interfere with host reproductive capacity, or the survivorship and reproductive success of other microbes in the community. As will become clear, the only indirect effects that influence microbial recruitment and persistence from one generation of hosts to the next are competition for space within hosts and the relative abundance of microbial taxa. Simply put, we assume that the ecological and evolutionary processes that operate on hosts and their microbiomes are neutral; in this regard, our framework is analogous to neutral theories in evolutionary biology [36,37], ecology and biodiversity [34]. We expand on this analogy later, but for now, we note that neutral theories provide parsimonious accounts of the types of patterns that can emerge in complex systems, they serve as null models for statistical hypothesis tests, and they provide platforms upon which we may construct more elaborate representations of these same systems [38].

In this framework, hosts reproduce asexually in discrete generations, following a neutral Wright-Fisher process [39,40], where each individual in a succeeding generation chooses a parent randomly from the preceding generation. Hence, with a population of hosts of constant size *N*, all asexual individuals will share a common ancestor after 2*N* generations, on average. In our models, asexual reproduction is a computational convenience, and can be replaced with sexual reproduction without changing the essential patterns that we observe.

**Fig 1:**
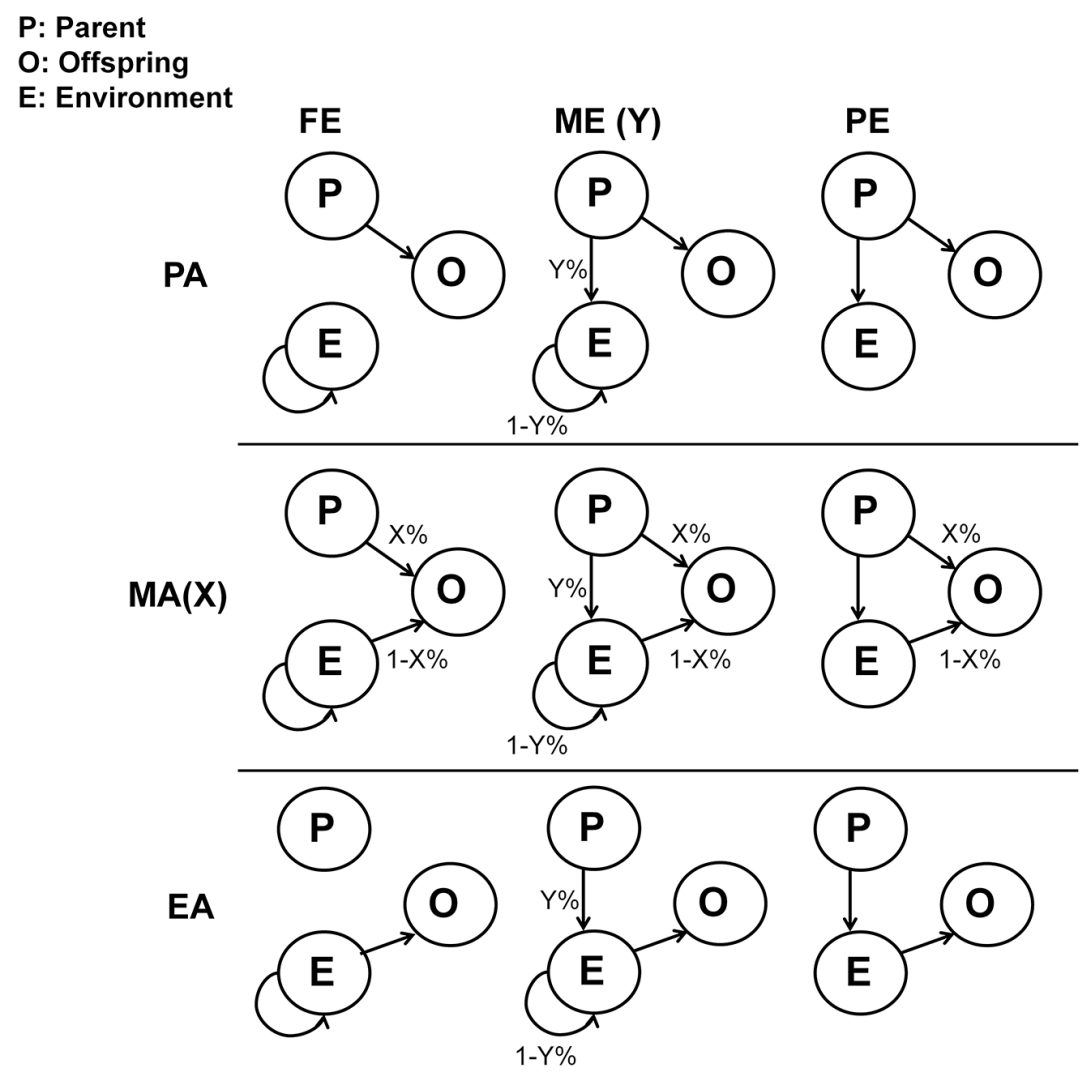
The relationship among parents, offspring and environment in different environmental and acquisition models. The columns represents different models of the environmental pool; the rows represents different models of microbiome acquisition. Arrows indicate how the environmental community is assembled for each new generation, and how offspring acquire their microbiomes. The values of *y*% and *x*% represent the percentage of host contribution under mixed environment (ME), and the percentage of parental inheritance under mixed acquisition (PA), respectively.

We model how hosts acquire their microbiomes in three ways (Fig 1). First, under a strict “parental-acquisition” (PA) process, all hosts acquire their microbial communities directly from their parents. Second, with strict “environmental-acquisition” (EA), hosts acquire their microbiomes solely from the environment. Between these two extremes, we also allow a third “mixed-acquisition” (MA*_x_*) process, whereby hosts acquire some percentage, *x*%, of their microbiomes from their parents and (100-*x*)% from the environment. MA_0_ is exactly equivalent to EA, and MA_100_ to PA; as such, EA and PA designate boundary conditions of the ecological processes that mediate microbial acquisition in hosts.

It is worth pausing at this point to clarify what we mean when we say that hosts acquire their microbiomes from their “parents” or their “environments”. Our models do not explicitly take account of the life events–illness, infections, changes in environments or diets–of each host within a generation, nor does it consider microbial fluxes within the lifespan of each host. Instead, the microbial composition of each host is essentially measured as an aggregate over the single generation that the host exists. Consequently, when we quantify the percentage of microbes from parents and environment using, say, MA_10_, we mean that over the life of the host, 90% of its microbes come from the environment and 10% from its parent. In our models, it is possible that the parental contribution happened in the first 10% of the host’s life, or it may be that over the entire lifespan of the host, there was an ongoing contribution by the parent that amounted to 10% of the microbial composition.

Since we allow hosts to recruit microbes from an “environment”, we need to define how the microbial content of this environment is constituted. In simulations, we characterize microbial composition using a distribution of taxa’ relative abundances. We propose three processes that determine the composition of the pool of microbes available for recruitment. First, we assume that the environment has a microbial composition that remains fixed over time. For the “fixed environment” (FE), all taxa are present in the environment throughout the simulation, and are available to every generation of hosts. The second process we propose involves a changing environmental microbial profile, whereby the relative abundance of each microbial taxon available to the hosts in a given generation, is an aggregate of their abundances from all hosts of the preceding generation. Under this “pooled-environment” (PE), microbial composition is reflection of what was present in the parents of the current generation of hosts. A third, intermediate, process is a combination of the previous two “environments”: the environmental microbial pool available for recruitment contains a percentage, *y*%, from the parental pool of microbes, and (100-*y*)% of microbes from the fixed environment. Under this “mixed environment” (ME*_y_*), the proportion of contribution from host microbiomes is given by *y*. As with our acquisition models, ME_0_ and ME_100_ are equivalent to the boundaries FE and PE, respectively.

Our framework allows us to combine different host-acquisition processes with different ways of constructing the pool of available microbes in the environment. Conceptually, each of these combinations is a particular neutral model, capturing some of the elements previously discussed in the literature. For instance, PA or MA*_x_* incorporate the phylogenetic dependencies that Yeoman et al [31] discuss, and EA x PE is equivalent to what Costello et al [30] call dispersal limitation, whereby the local host community influences microbial composition. It is worth noting that the combinations PA x (FE, ME_y,_ PE)–read as “PA in combination with FE, with ME_y_ or with PE”–will give identical results. This is because, in all cases, the environment contributes nothing to host microbial content (see the first row in Fig 1).

We model the construction of the microbial community in each host by competitive random sampling with replacement. Under this process, each host allows only a fixed and limited number of microbes to populate its microbiome. If microbial acquisition occurs under EA, each host samples randomly from the available pool of taxa according to the relative abundance of each taxon in the environment. In the case of MA*_x_*, *x*% of microbes are selected from the parent and (100-*x*)% from the environment. If hosts acquire their microbial taxa under PA, then all microbes are inherited from the hosts’ parents, although the relative abundance of each taxon fluctuates multinomially. By constructing microbial communities in this way, we allow stochastic factors and indirect competition to modify taxon composition within and between hosts, as proposed by Costello et al [30].

By simulating combinations of PA, MA*_x_* and EA against FE, ME_y_ and PE forward in time over many host generations and over a range of conditions, we are able to recover data on the behavior of individual microbial taxa, as well as a variety of summary statistics, including the expected time it takes individual taxa to invade all hosts or go extinct in the host population, and the trajectories of microbial taxonomic richness (measured simply as the total number of microbial taxa) and microbial taxonomic evenness (measuring the similarity in the frequency of each taxon), microbial diversity within hosts (α-diversity), inter-host variation in microbial composition (β-diversity) and the aggregate microbial diversity from all hosts in the population (γ-diversity). Here, we report only on the latter three measures of diversity.

## Results

### Relative abundances of microbial taxa in the host population

**Fig 2:**
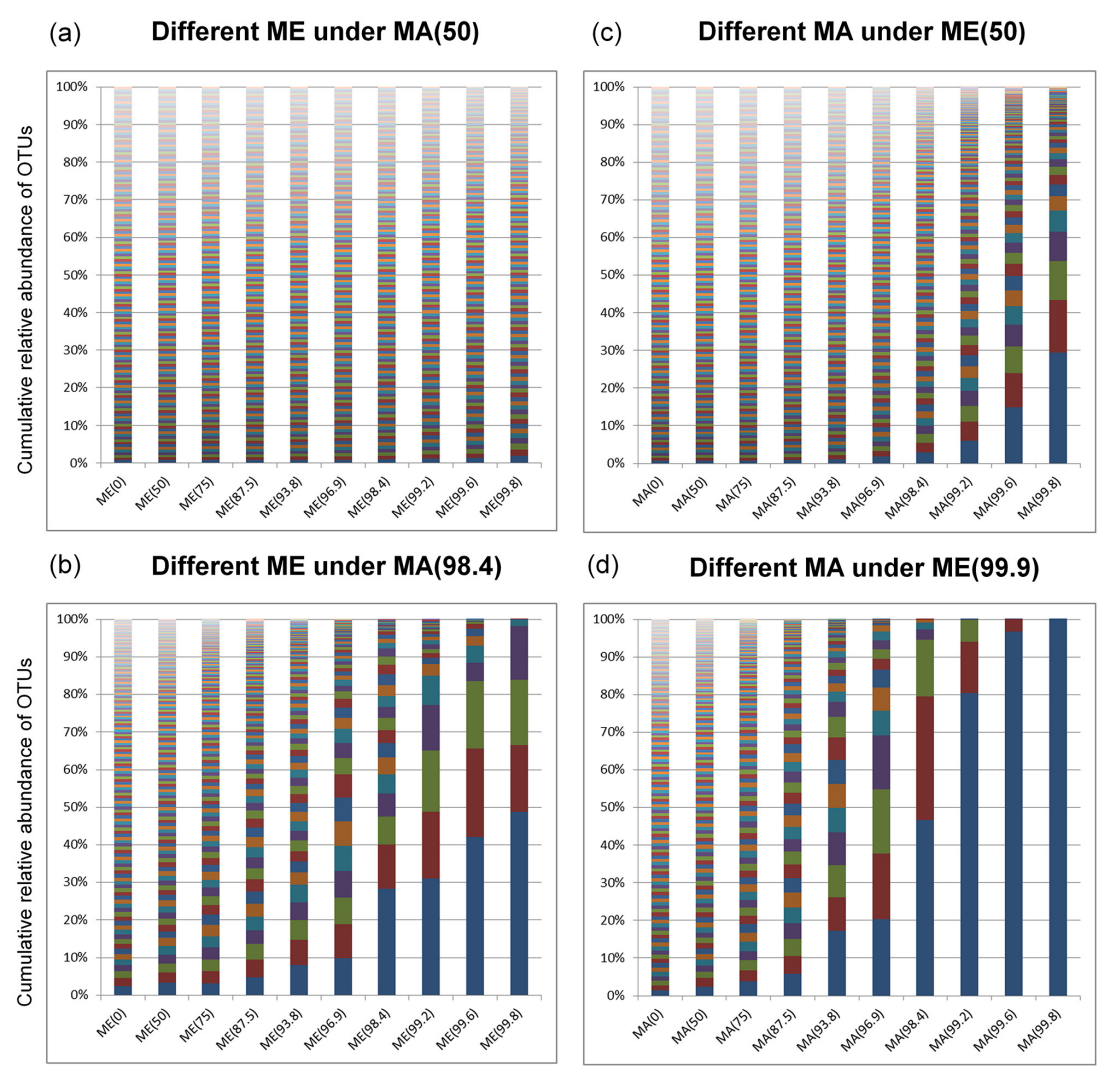
Distribution of relative taxon abundance within the host population under combinations of MA and ME. (a) distribution of relative taxon abundances with different parental contributions to the environment (ME), when the proportion of direct parental acquisition of microbes is low (i.e., MA(50) or 50% parental acquisition); (b) distribution of relative taxon abundances with different parental contributions to the environment (ME), when the proportion of direct parental acquisition of microbes is high (i.e., MA(98.4) or 98.4% parental acquisition); (c) distribution of relative taxon abundances with different proportions of microbes directly acquired from parents (PA), when the proportion of parental microbial contribution to the environmental pool is low (i.e., ME(50) or 50% parental contribution); (d) distribution of relative taxon abundances with different proportions of microbes directly acquired from parents (PA), when the proportion of parental microbial contribution to the environmental pool is high (i.e., ME(99.9) or 99.9% parental contribution).

**Fig 3:**
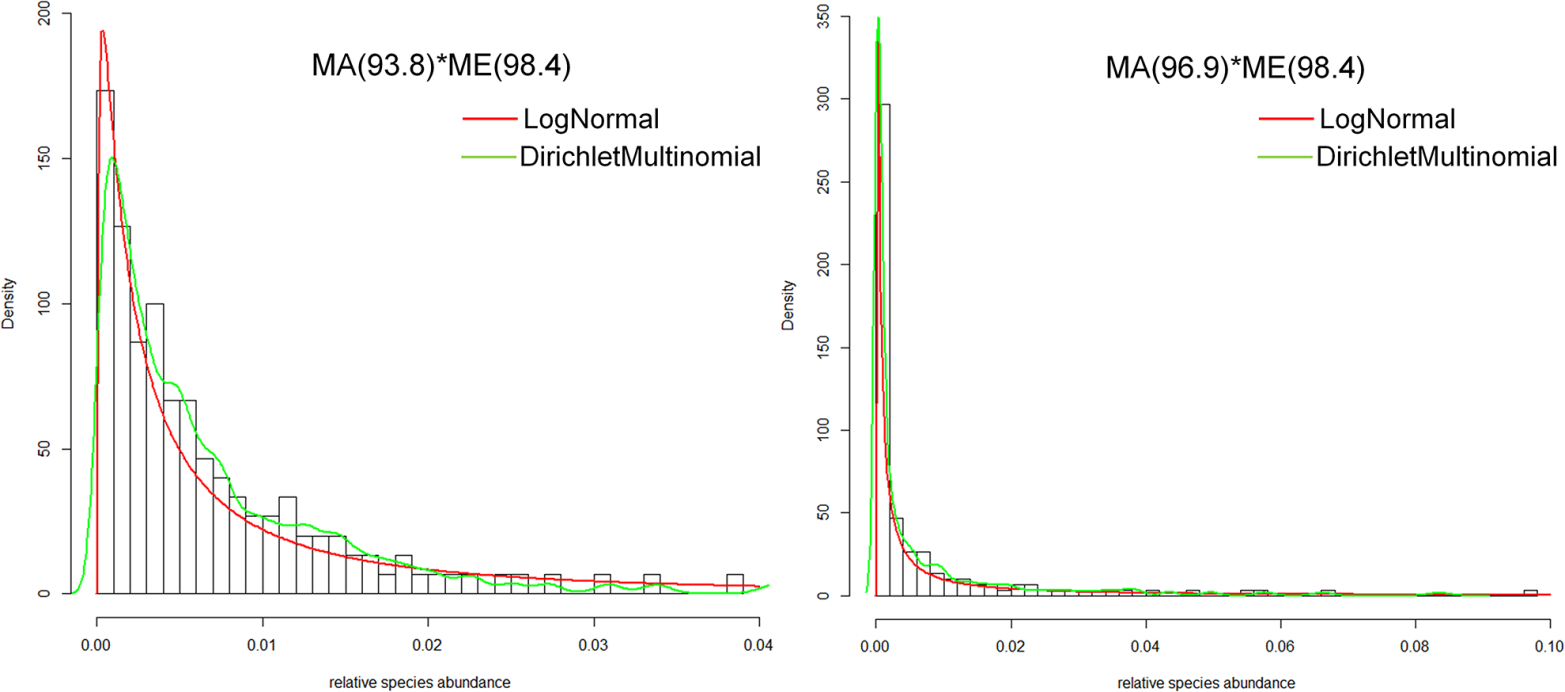
Log-normal and Dirichlet-Multinomial fitting of simulated abundance distributions. Relative frequency histograms represent the abundance distributions of simulated microbial communities under two mixed models. The red lines are the probability density curves of fitted log-mormal distributions. The green lines are smoothed density curves from Dirihchlet-Multinomial distributions.

Microbial diversity within the host population is a function of the proportion of microbes that parents contribute directly to offspring and the proportion they contribute to the environment. Fig 2 illustrates how population-level taxon abundances change under various combinations of these proportions. In our simulations, the distributions of taxon abundances under high levels of parental contributions are skewed, and may be approximated by commonly-applied distributions, including the log-normal distribution and the Dirichlet multinomial (DM) distribution [41] (Fig 3; the DM distribution has the advantage of allowing α-, β- and γ-diversities to be simulated–see S2 Fig). The ability to recover skewed abundance distributions is interesting, because we begin our simulations with a uniform distribution of microbial taxa, and we retain this uniform distribution in the fixed environment throughout the evolutionary history of the host population. Consequently, the emergence of dominant and rare taxa is a consequence of repeated parental contributions either directly to the next generation of hosts or indirectly to the environment.

In fact, all simulations in which there was complete parental acquisition of microbes (i.e., PA) resulted in the loss of all but one microbial taxon in the host population. Similarly, when the environment was reconstituted each generation exclusively with microbes from the parents (i.e., PE), the same pattern was observed with only a single microbial taxon remaining. These result are consistent with predictions made under neutral models of community ecology [42], and highlight the strong depressive effect of parental transmission, either directly from parent to offspring or via parental contributions to a local pool of microbes, on population-level microbiome diversity.

With EA x FE, microbes are obtained randomly from a fixed environment that persists over the evolutionary history of the hosts; unsurprisingly, the host population retains all microbes found in the environment. Interestingly, when microbes are obtained both from parents and a fixed environment (MA x FE), we still see the persistence of all or almost all microbes in the host population (see Fig 2A and 2B, first column of each bar chart; ME(0) is equivalent to a fixed environment with no microbial contributions from parents). This is true even when the proportion of microbial taxa that an individual host acquires from the fixed environment at each generation is very small, on the order of 0.001. Therefore, a very small contribution from a constant environmental source of microbes is sufficient to retain high levels of microbial diversity in the host population.

### Measures of diversity

Microbial diversities are frequently measured in three ways: α-diversity, β-diversity, and γ-diversity. Our simulations indicate that all three measures depend on the percentage of parental contribution to offspring microbiomes and the composition of the environmental microbial pool (see S1–S3 Tables for simulation means and standard deviations).

**Fig 4:**
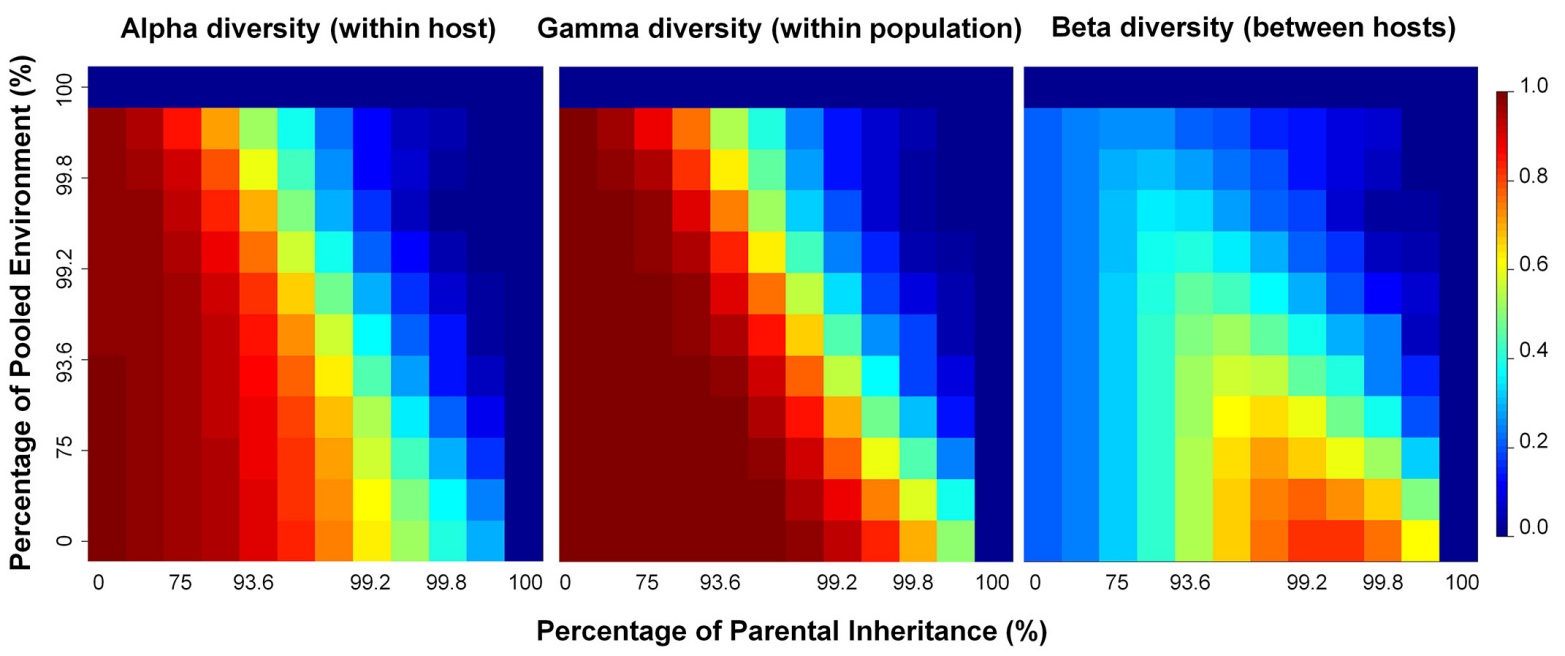
Heatmap of α-, γ-, β-diversity with different combinations of acquisition models and environmental models. For each heatmap, the proportion of parental inheritance increases and environmental acquisition decreases from left to right; similarly, the environment microbial composition is increasingly derived from the parental microbiomes as we move from the bottom of each heatmap to the top. Each diversity value is averaged from 10 independent simulations.

Under our neutral model, in which the absence of host sub-population structure means that all hosts sample their microbes from the same environment, α- and γ-diversities remain high, and β-diversity remains low, for a large part of the range of direct or indirect parental contributions (i.e., to offspring or to the environment, respectively). Nonetheless, at high values of parental contributions, there are discernible differences in diversities, and we have also focused our simulations in these areas (Fig 4; see S4–S6 Tables for simulation means and standard deviations). In general, α-diversity (average diversity within hosts) and γ-diversity (overall diversity within the entire population of hosts) increase as we increase the fixed environmental contribution because a fixed environment helps maintain a uniform distribution of taxon abundances and delays the loss of microbial taxa during evolution. Conversely, when hosts acquire increasing proportions of their microbiomes from their parents directly, or indirectly from a pooled environment, the variation of taxon abundance increases and taxon richness tends to decrease, thus lowering both α- and γ-diversities (Fig 4A and 4B; S1, S2, S4 and S5 Tables). Inter-host variation in microbial composition, or β-diversity, also depends on the degree of parental inheritance, and the ratio of fixed-to-pooled environmental components (Fig 4C; S3 and S6 Tables).

Under the combination of PA x (FE, ME or PE), β-diversity tends to zero, because all hosts descend from a single common ancestor and, as noted above, only a single microbial taxon remains in all hosts. When we have the parental microbiome as the only source of microbiomes in the next generation, ultimately, all lineages will have acquired their microbiomes from the most recent common ancestor (MRCA) of the population of hosts. Additionally, from one generation to the next, stochastic sampling of microbes over evolutionary time will result in the loss of all but one microbial taxon. Interestingly, with a high percentage of environmental acquisition, β-diversity is also relatively low, because all hosts acquire a large proportion of their microbial taxa from the same environmental pool, and consequently, will tend to acquire the same set of taxa. As noted above, the highest β-diversity occurs in a relatively narrow range of values of pure parental acquisition (between 87–99% of direct parental transmission; S6 Table).

If we focus on the relationship between β-diversity and the environmental pool, we see that its behavior is similar to that of α- and γ-diversities: it decreases as we increase the pooled environmental contribution to offspring microbiome. This is because a pooled environment, with contributions from the parental generation, tends to give rise to a non-uniform distribution of microbial taxa. As the degree of parental contribution increases, the environmental community will be dominated by few highly abundant species which are likely shared by most or all hosts within the population, accounting for high between-host similiarity in microbial composition (Fig 4C; S3 and S6 Tables).

It is important to note that our simulations have not been performed with inference or prediction in mind: the number of hosts, the number of microbes, and the number of taxa in our simulations are not necessarily equivalent to those of real-world microbial communities and their hosts, nor have we necessarily chosen the appropriate diversity indices or taxonomic resolution to optimize prediction/inference. Nonetheless, it is helpful to examine how the simulated values of diversity compare to empirical observations, and what these comparisons might tell us about the evolutionary processes that are acting on microbiomes. As an example, we used genus-level taxonomic data from the NIH Human Microbiome Project (HMP) [43], specifically, a table of relative abundance found in different compartments of the human body (http://www.hmpdacc.org/HMSMCP/; see S1 Dataset). Several large samples from the anterior nares, vaginal posterior fornix, stool, buccal mucosa, tongue dorsum and supragingival plaque were chosen to calculate α-, β- and γ-diversities on genus level (Table 1).

**Table 1:** Diversities of microbiota associated with different human body sites.

**Body Site**	**α-Diversity** ^1^	**γ-Diversity** ^2^	**β-Diversity**	**Number of samples**
Posterior Fornix	0.041±0.067	0.097	0.175±0.294	38
Stool	0.267±0.093	0.381	0.464±0.213	94
Supragingival Plaque	0.457±0.061	0.531	0.455 ±0.136	85
Tongue Dorsum	0.399±0.051	0.454	0.387±0.137	87
Anterior Nares	0.223 ±0.071	0.318	0.451 ±0.210	69
Buccal Mucosa	0.265±0.079	0.315	0.318 ±0.135	77

^1^The α- and β- diversities are mean values and standard errors averaged over all samples or over all pairs of samples.

^2^The γ- diversity is a single value because it is calculated with a large sample that is pooled from all the samples within the same body site; hence no replicates or ranges are available.

Values of α- and γ-diversity obtained from all sampled sites of the human microbiome are low, in comparison to most of the values we obtained in our simulations. In fact, human microbiome diversities are generally lower than those of other non-human primates [44,45]. If we compare the empirical diversities to those obtained in our simulations (S1–S6 Tables), we would have to posit very high parental contributions, both direct and indirect (>90%), to account for the α- and γ-diversities across all human body sites. In contrast, values of β-diversity appear to provide a little more discrimination amongst body sites: the site with the lowest β-diversity is the vaginal posterior fornix, and its value is consistent with a very low degree of direct parental contribution in our simulations (approximately between 0–15%). The β-diversities at other sites appear to suggest higher levels of parental contribution (again, >90%). In the next section, we discuss the implications of these results, as they relate to human microbiome evolution and how the neutral model may be used to construct hypotheses about relevant evolutionary and ecological processes.

## Discussion

In this paper, we introduce a simple and flexible framework to model the evolution of microbiomes within a population of hosts, which takes account of different modes of microbiome acquisition and environmental microbial composition. Under our neutral model, microbiome composition is affected by sampling effects. Stochastic changes in microbial abundances may affect the persistence of microbial taxa in the microbiome over one or a few generations (i.e., ecological drift), or over many generations. The latter may occur because host lineages die out; when this happens, changes in microbial abundance across the whole population of hosts are essentially equivalent to changes in allele frequencies (i.e., genetic drift).

The constitution of the microbial community in the environment also plays a considerable role in determining the ultimate fate of microbial taxa within host microbiomes. With a fixed environment, when there is a constant pool of the same microbial taxa from one generation of hosts to the next, microbial taxa never go extinct from the host population as long as hosts obtain some fraction of their microbiome from the environment. This is true, even when that fraction that the environment contributes to each host’s microbiome is very small (e.g., 0.1%). In contrast, when the environmental composition of microbes reflects the microbial content of the hosts in previous generations (i.e., PE, the “pooled” environment in our model), microbial diversity of the environment shrinks, as does the diversity of host microbiomes. Therefore, the extent to which parents contribute to the microbiomes of their offspring (either directly or through their contributions to the pooled environment) plays a crucial role in shaping microbiome diversity and constitution. In our simulations, values of α- and γ- diversities are at their lowest when parental contribution to the microbiome is high. Inevitably, microbial taxa are lost from the population as host lineages are lost.

Thus, under our neutral model, it is possible to recover skewed microbial abundance distributions reminiscent of those obtained with real data, despite a fixed environmental component that remains uniform and constant throughout our simulations. Increasing skewness–essentially, decreasing eveness–is obtained as we increase the degree of parental inheritance. Of course, we don’t claim any deep insight here: no one should be surprised that we are able to recover skewed abundance distributions with our models, because there is a large body of literature on the mechanisms–both neutral or otherwise–that may lead to the emergence of skewed abundance distributions (see [46] for an excellent synthesis). Our results reinforce what others [34,47,48] have found, by adding yet another neutral mechanism to account for the emergence of skewed abundance distributions.

Our framework includes sampling effects on an undivided host population, which evolves under a Wright-Fisher process. Consequently, our models have some points of similarity with those that have been developed in population genetics. For instance, Orive et al [49] analyze the evolutionary dynamics of endosymbionts using a discrete-time Moran population genetic model. In their model, endosymbionts are acquired either vertically, passed on from parent to offspring, or horizontally from the environment. This corresponds to our MA x FE model and, in agreement with our results, Orive et al find that increasing the environmental contribution of endosymbionts to host cells results in greater diversity within cells and less diversity between cells.

Our models do not include any mutational process or speciation acting on the microbes, as time moves forward. In reality, of course, microbes acquire mutations in their genomes at a rapid rate, but the measures of diversity we use in our analyses capture differences in taxonomic composition, not genetic diversity. In our models, it is implied that no cladogenetic events have occurred over the course of the simulations.

The models presented here provide an opportunity to construct hypotheses, and make qualitative predictions, about the patterns of diversity we can expect to find in different biological situations. For example, the effects of “pure” pooled versus fixed environments on microbiomes can be found in a comparison of social and solitary bees. Social bees exhibit behaviors that are likely to result in the transmission of microbes from a microbial pool within the colony [50]. In contrast, solitary bees acquire their microbiomes from the environment, through feeding or burrowing. Our model would predict that social bees would have lower α-diversity and lower taxonomic richness than solitary bees. This is consistent with the results obtained by Martinson et al [51] who surveyed the microbiomes of eusocial bee species *Apis* spp. and *Bombus* spp. and non-social bees (11 species) and wasps (3 species): they found depauperate microbiomes in social bees compared to non-social bees.

Whereas it is reassuring to obtain empirical corroboration for our models, arguably neutral models are most useful when real-world observations run counter to predicted outcomes. Falsification of neutral models provides a justification for augmenting these models to include additional processes that account for the phenomena under study. In this regard, our analysis of the Human Microbiome Project data is instructive. As we have noted above, our simulations should not be used for inference, and we should be cautious about reading too much into the comparisons between empirical and simulated patterns of diversity. Nonetheless, at least for some sites, i.e., the stool, the tongue dorsum, the supragingival plaque, the anterior nares and the buccal mucosa, the low empirical values of α- and γ- diversities appear to point consistently to a high level of parental inheritance when compared against values obtained in our simulations. There is evidence that the human microbiomes at various sites are seeded at birth by the mother, particularly if this birth is through the vaginal tract [52]. There is also reasonably strong evidence that families share microbes to a greater extent than unrelated individuals in a population [53], and at least in some human populations, mothers share more microbes in common with their offspring than with unrelated children [54]. It is not clear, based on the studies that have been done to date [55], whether the values of direct or indirect parental contribution we obtain when we compare empirical and simulated diversities are significantly higher than would be obtained in real populations, but we expect that the intuition of mosts microbial ecologists is that percentages of direct and pooled parental contributions > 90% are likely to be too high. Putting to one side the caveats about inference, we accept that while this intuition does not constitute evidence against the neutral model, it is likely to engender scepticism about the model’s correctness. If it is, in fact, true that direct or indirect parental contributions to the next generation’s microbiomes are not as high as our simulations suggest, how do we account for the apparent depression in α- and γ-diversities, and elevation of β-diversities at these sites? One hypothesis that explains low α- and γ-diversities, and high β-diversities, and does not require the action of non-neutral processes, is the existence of local host subpopulations. The existence of subpopulations of hosts, with limited immigration and sharing of microbes between subpopulations, is likely to give the appearance of high parental contribution from one generation to the next. Certainly, this is a plausible explanation for patterns of microbiome diversity in the oral cavity (i.e., the buccal mucosa, tongue dorsum and supragingival plaque) and stool samples, because of the likely influence of familial [53] or cultural dietary preferences/practices [56] or lifestyles [57] on these microbiomes. A similarly explanation may account for patterns of microbiome diversity of the anterior nares.

The vaginal posterior fornix presents an interesting contrast to the other body sites because the α- and γ-diversities suggest high parental contributions (although they cannot distinguish between direct or indirect contributions), whereas β-diversity suggests a low direct parental contribution. This inconsistency may again cause us to reject the neutral model in favor of an alternative explanation, but in this case, subpopulation structure may play a minor role relative to selection for a vaginal microbial community that is common amongst hosts. Such a selective filter is likely a consequence of a complex suite of factors including host immune defences, hormonal cycling, pregnancy, and the presence of apparently beneficial microbial species (e.g., *Lactobacillus* spp.) [58]. This hypothesis explains both the high level of α- and γ-diversity (i.e., a few abundant species with many rare species), and the low β-diversity.

For the human microbiome, neutral models have the potential to help identify additional processes that may account for patterns of diversity. As noted, of the two processes identified above–host subpopulations and selective filters–the former still remains part of an underlying neutral process, and a plausible extension to the neutral framework presented here. Rejection of a simple neutral model therefore allows us to identify incremental additions that may increase explanatory power.

Another example of empirical data that appears to contradict the expectations of our models is the comparison of microbiome diversities in high microbial abundance (HMA) and low microbial abundance (LMA) sponges [59]. HMA sponges have large numbers of associated microbes, in contrast to LMA sponges. Additionally, researchers have shown that microbial diversity in LMA sponges is lower than that of HMA sponges [60,61]. Based on our results, we would predict that there is a greater degree of vertical transmission in LMA sponges, but it turns out that this is not the case: Schmitt et al [62] have found that “vertical transmission, as a mechanism to obtain bacteria, seems to occur mainly in HMA sponges”. Giles et al [60] propose two possible reasons to account for the low diversity in LMA sponges. First, there may be selective filters that permit only certain microbial taxa to colonize the sponges; second, the initial colonization event is stochastic, but serves to constrain or exclude successive colonizations. As with the human microbiome data, the sponge example is important because it does not rely *a priori* on non-neutral processes to account for the low diversity in LMA sponges; instead, selection (or other ecological and/or evolutionary processes) is invoked only after it is shown that vertical transmission in LMA sponges is unlikely, thus indicating that our neutral models are an inadequate explanation for the observed data.

Riffing on the theme that “Essentially, all models are wrong, but some models are useful” [63], Hubbell, writing about models in community ecology, says “Probably no ecologist in the world with even a modicum of field experience would seriously question the existence of niche differences among competing species on the same trophic level” [64]. But, he continues, “[Neutral theory] begins with the simplest possible hypothesis one can think of … and then adds complexity back into the theory only as absolutely required to obtain satisfactory agreement with the data”. We agree with Hubbell: to paraphrase, given what we know about the interplay between hosts, their microbial communities, and the environment, we would hesitate to put money on the table and bet that many microbiomes have evolved under the simplest neutral models that we have constructed here. But we would be equally hesitant betting in favor of the null hypotheses evaluated in statistical tests of significance. The value of these hypotheses resides not in their rightness or wrongness but in their ability to protect against overconfidence in our favorite, more complex model. Whereas it is true that biological processes are frequently complex, Occam’s Razor dictates that we construct as simple explanations (or models) as possible. In this way, we remain vigilant against the addition of unnecessary and unjustifiable complexity. Much as we do with statistical hypothesis tests, we accept stronger alternative explanations only when we are sufficiently confident that our neutral hypotheses are unlikely.

This is not to say that neutral models only serve as strawmen; in molecular evolution, for instance, neutral models are frequently effective at explaining molecular variation [65]. And even in cases when the assumption of neutrality is questionable, the use of neutral models of substitution applied in molecular phylogenetics does not appear to jeopardize the accuracy of tree reconstruction [66]. Consequently, without taking account of the evolutionary processes of mutation, speciation, selection or recombination, or the ecological processes that operate in the context of spatial, environmental, and temporal heterogeneity, what we have developed is a framework on which we can begin to evaluate empirical patterns of diversity, and where necessary, add more elaborate ecological and evolutionary scenarios. We believe that even this simple framework, devoid as it is of all the embellishments afforded by evolution and ecology, can serve a useful purpose: it is a suitable staging ground on which we can construct null models of microbiome diversity in populations of hosts and it allows us to make strong, testable predictions.

## Materials and Methods

Simulated host populations consisted of a fixed number of virtual host individuals (*N* = 500). Each host was allocated a virtual microbiome with a limited capacity or "slots" of microbes (*n* = 1000). The environmental pool consisted of 150 microbial taxa. Large number of hosts (*N* = 2000), microbes (*n* = 100000) per host and microbial taxa (*m* = 500) were also simulated with our neutral model, and similar patterns of diversity were observed (S1 Fig).

The microbiomes of the initial generation of host individuals were seeded randomly, with bacteria sampled from a uniform distribution of taxon abundances. We used an initial uniform distribution of taxa because we wanted to ascertain whether the equilibrium distribution of abundances obtained at the conclusion of our simulations would recover patterns seen in natural microbiomes.

For each subsequent generation, the microbiome of each individual host was simulated by populating each of the available "slots" in the individual's microbiome by sampling microbial taxa with replacement (multinomial choice) from either the environment (with probability given (1-*x*)) or from the microbiome of a "parent" host individual (selected with uniform random probability from the population of the previous generation).

When sampling from a parental/environmental microbial community, the probability that the new host microbiome will acquire a particular microbial taxon is given by the relative abundance of that taxon within the community (see below for details on how environmental microbial taxon abundances were calculated).

The probability, *x*, that a particular "slot" in a new individual host's microbiome was occupied by a microbial taxon sampled from a randomly selected parent was varied across simulations. Two sets of simulations were performed: (1) *x* and *y* varied linearly, between 0 and 1, with increments of 0.1 (see S1–S3 Tables for means and standard deviations of diversities); and (2) with values of *x*, *y* ∈ (0.0, 0.5^[0,1,2…10]^) (see S4–S6 Tables for diversities). When *x* = 0.0, a host’s microbiome was sampled directly from the environment, i.e., the probability of a microbial taxon being selected was equal to the relative frequency of the microbial taxon in the environment. When *x* = 0.5^0^ = 1, a host's microbiome was sampled multinomially from the microbiome of its "parent" who was selected with uniform random probability from the previous generation. Similarly, when *y* = 0.0, there is no contribution from the previous host generation to the environmental microbial resource, whereas when *y* = 0.5^0^, all microbes are replaced each generation by the pool of microbes resident in the hosts of the previous generation.

Three different models were used to model the relative abundance of taxa in the environment. First, for FE ("fixed environment"), the abundances of the microbial taxa in the environment were fixed to the initial uniform distribution and did not vary over the course of the simulation. Second, under PE ("pooled environment") the abundances of the microbial taxa in the environment were composed of the pooled microbiomes of all the hosts of the previous generation, i.e., by summing over the abundances of the respective microbial taxa in hosts’ microbiomes, and renormalizing to relative abundances. Third, under ME*_y_* (“mixed environment”), the abundances of the microbial taxa in the environment were calculated by combining the fixed environment and pooled environment with *y*% derived from the pooled environmental component.

A particular simulation regime consisted of a distinct combination of pooled/fixed environmental ratios and environmental factors. A total of ten replicates were run under each simulation regime. The plots of γ-diversity were inspected, and simulations were halted when these stabilized. The number of generations for each simulation varied between 10^4^ and 10^6^ generations.

After each generation was simulated, the diversities of microbiomes are measured by scaled Shannon-Wiener index (α-diversity and γ-diversity) or Bray-Curtis dissimilarity index (β-diversity). The scaled Shannon-Wiener index is calculated as $-\frac{\sum_{i=1}^{R}p_{i}ln(p_{i})}{\ln R}$, where *R* represents the total number of taxa and *p_i_* represents the relative abundance of *i*th taxon within the community. The calculation of the Bray-Curtis index is given by the formula: $\frac{2}{(n-1)n}*\;\sum_{i=2}^{n}\sum_{j=1}^{i-1}\sum_{k=1}^{m}\frac{{|p}_{ik}-p_{jk}|}{2}$, where *n* represents the total number of hosts in the population, *m* represents the total number of microbial taxa within the host population, and *p*_*ik*_ and *p*_*jk*_ represents the relative abundance of *k* ^th^ taxon within the community of host *i* and host *j*.

Empirical data from the human microbiome were obtained from the website of the NIH Human Microbiome Project (http://www.hmpdacc.org/HMSMCP/). The original community profiling data is a table of relative abundances for each of 690 samples and 718 taxa of bacteria and archaea (from 2 kingdoms to 397 species). As is described on the HMP website, all the samples were collected from 16 body sites from 103 healthy humans and processed with Whole Genome Shotgun sequencing. Specific information of each sample is available on the website (http://www.hmpdacc.org/HMIWGS/all/). We selected data of microbial communities associated with anterior nares, buccal mucosa, supragingival plaque, stool, tongue dorsum and vaginal posterior fornix because of their large numbers of samples, and removed the replicated samples from the same human subject for each body site (see S1 Dataset). The HMP data provides relative abundances for different taxonomic levels. We calculated diversities for all taxonomic levels ranging from species to kingdom, and found that the values of diversities for genus, family and order were similar. Consequently, we chose to use genus-level diversities. The relative abundances of genera for each site and all samples is given in S1 Dataset.

Fitting simulated results into log-normal and Dirichlet-multinomial distributions was performed in R with methods “fitdistr” of package MASS and “dirmult” of package dirmult.

All simulations were carried out using Python scripts and Java programs, available from https://github.com/qz28/microbiosima.git

Java code: https://github.com/qz28/microbiosima/tree/master/java

Python scripts: https://github.com/qz28/microbiosima/tree/master/python

## Supporting Information

S1 Table α-diversity under different combinations of acquisition and environment models with linear scales for MA(X) and ME(Y). (DOCX)

S2 Table γ-diversity under different combinations of acquisition and environment models with linear scales for MA(X) and ME(Y). (DOCX)

S3 Table β-diversity under different combinations of acquisition and environment models with linear scales for MA(X) and ME(Y). (DOCX)

S4 Table α-diversity under different combinations of acquisition and environment models with power scales for MA(X) and ME(Y). (DOCX)

S5 Table γ-diversity under different combinations of acquisition and environment models with power scales for MA(X) and ME(Y). (DOCX)

S6 Table β-diversity under different combinations of acquisition and environment models with power scales for MA(X) and ME(Y). (DOCX)

S1 Dataset Relative abundance data of empirical human microbial communities associated with anterior nares, buccal mucosa, stool, posterior fornix, tongue dorsum and supragingival plaque from Human Microbiome Project. (XLSX)

S1 Fig Heatmap of α-, γ-, β-diversity with different combinations of acquisition model and environmental model (Simulated with 2000 hosts, 100000 microbes per host, and 500 microbial taxa until 15000 host generations). The diversity patterns are also represented with heatmaps in a similar way (the proportion of parental inheritance = 1–0.5*^x^* with *x* ranging from 0 to 20; the proportion of pooled environmental component = 1–0.5*^y^* with *y* ranging from 0 to 20), and a similar pattern were still observed regardless of the increasing numbers of hosts, microbes and microbial taxa.
(TIF)

S2 Fig Heatmap of α-, γ-, β-diversity reconstructed from Dirichlet-Multinomial distribution. The same log-scales as in Fig 4 is used, and each square represents a re-estimated diversity value from a fitted Dirichlet-Multinomial distribution with ten replicates.
(TIF)
