# Supplementary material for: Neutral Models of Microbiome Evolution
Source: PLoS Comput Biol. 2015 Jul 22;11(7):e1004365. doi: 10.1371/journal.pcbi.1004365 (PMC4511668; doi:10.1371/journal.pcbi.1004365)
Supplement: S1 Table — (DOCX) [file pcbi.1004365.s001.docx]

**S1 Mean α-diversities ± standard deviations under different combinations of acquisition and environment models**

|  | **EA** | **MA(10)** | **MA(20)** | **MA(30)** | **MA(40)** | **MA(50)** | **MA(60)** | **MA(70)** | **MA(80)** | **MA(90)** | **MA(99)** | **PA** |
| --- | --- | --- | --- | --- | --- | --- | --- | --- | --- | --- | --- | --- |
| **PE** | 0.000±0.000 | 0.000± 0.000 | 0.000± 0.000 | 0.000± 0.000 | 0.000± 0.000 | 0.000± 0.000 | 0.000± 0.000 | 0.000± 0.000 | 0.000± 0.000 | 0.000± 0.000 | 0.000± 0.000 | 0.000± 0.000 |
| **ME**  **(99)** | 0.983±0.000 | 0.983± 0.000 | 0.982± 0.000 | 0.981± 0.000 | 0.979± 0.000 | 0.976± 0.000 | 0.971± 0.001 | 0.961± 0.001 | 0.940± 0.002 | 0.870± 0.008 | 0.290± 0.052 | 0.000± 0.000 |
| **ME**  **(90)** | 0.985±0.000 | 0.984± 0.000 | 0.984± 0.000 | 0.983± 0.000 | 0.982± 0.000 | 0.979± 0.000 | 0.976± 0.000 | 0.970± 0.000 | 0.957± 0.000 | 0.921± 0.000 | 0.573± 0.020 | 0.000± 0.000 |
| **ME**  **(80)** | 0.985± .000 | 0.984± 0.000 | 0.984± 0.000 | 0.983± 0.000 | 0.982± 0.000 | 0.980± 0.000 | 0.976± 0.000 | 0.970± 0.000 | 0.959± 0.000 | 0.925± 0.001 | 0.612± 0.024 | 0.000± 0.000 |
| **ME**  **(70)** | 0.985±0.000 | 0.985± 0.000 | 0.984± 0.000 | 0.983± 0.000 | 0.982± 0.000 | 0.980± 0.000 | 0.976± 0.000 | 0.971± 0.000 | 0.959± 0.000 | 0.926± 0.000 | 0.640± 0.007 | 0.000± 0.000 |
| **ME**  **(60)** | 0.985±0.000 | 0.984± 0.000 | 0.984± 0.000 | 0.983± 0.000 | 0.982± 0.000 | 0.980± 0.000 | 0.976± 0.000 | 0.971± 0.000 | 0.959± 0.000 | 0.927± 0.001 | 0.654± 0.006 | 0.000± 0.000 |
| **ME**  **(50)** | 0.985±0.000 | 0.985± 0.000 | 0.984± 0.000 | 0.983± 0.000 | 0.982± 0.000 | 0.980± 0.000 | 0.976± 0.000 | 0.971± 0.000 | 0.959± 0.000 | 0.927± 0.001 | 0.659± 0.004 | 0.000± 0.000 |
| **ME**  **(40)** | 0.985±0.000 | 0.985± 0.000 | 0.984± 0.000 | 0.983± 0.000 | 0.982± 0.000 | 0.980± 0.000 | 0.976± 0.000 | 0.971± 0.000 | 0.959± 0.000 | 0.928± 0.001 | 0.664± 0.009 | 0.000± 0.000 |
| **ME**  **(30)** | 0.985±0.000 | 0.985± 0.000 | 0.984± 0.000 | 0.983± 0.000 | 0.982± 0.000 | 0.980± 0.000 | 0.976± 0.000 | 0.971± 0.000 | 0.959± 0.000 | 0.928± 0.001 | 0.666± 0.007 | 0.000± 0.000 |
| **ME**  **(20)** | 0.985±0.000 | 0.985± 0.000 | 0.984± 0.000 | 0.983± 0.000 | 0.982± 0.000 | 0.980± 0.000 | 0.976± 0.000 | 0.971± 0.000 | 0.959± 0.000 | 0.928± 0.000 | 0.668± 0.009 | 0.000± 0.000 |
| **ME**  **(10)** | 0.985±0.000 | 0.985± 0.000 | 0.984± 0.000 | 0.983± 0.000 | 0.982± 0.000 | 0.980± 0.000 | 0.976± 0.000 | 0.971± 0.000 | 0.959± 0.000 | 0.928± 0.001 | 0.672± 0.007 | 0.000± 0.000 |
| **FE** | 0.985±0.000 | 0.985± 0.000 | 0.984± 0.000 | 0.983± 0.000 | 0.982± 0.000 | 0.980± 0.000 | 0.976± 0.000 | 0.971± 0.000 | 0.959± 0.000 | 0.928± 0.001 | 0.675± 0.006 | 0.000± 0.000 |
