# Supplementary material for: Neutral Models of Microbiome Evolution
Source: PLoS Comput Biol. 2015 Jul 22;11(7):e1004365. doi: 10.1371/journal.pcbi.1004365 (PMC4511668; doi:10.1371/journal.pcbi.1004365)
Supplement: S2 Table — (DOCX) [file pcbi.1004365.s002.docx]

**S2 Mean γ-diversities ± standard deviations under different combinations of acquisition and environment models**

|  | **EA** | **MA(10)** | **MA(20)** | **MA(30)** | **MA(40)** | **MA(50)** | **MA(60)** | **MA(70)** | **MA(80)** | **MA(90)** | **MA(99)** | **PA** |
| --- | --- | --- | --- | --- | --- | --- | --- | --- | --- | --- | --- | --- |
| **PE** | 0.000±0.000 | 0.000± 0.000 | 0.000± 0.000 | 0.000± 0.000 | 0.000± 0.000 | 0.000± 0.000 | 0.000± 0.000 | 0.000± 0.000 | 0.000± 0.000 | 0.000± 0.000 | 0.000± 0.000 | 0.000± 0.000 |
| **ME**  **(99)** | 0.999±0.000 | 0.998± 0.000 | 0.998± 0.000 | 0.998± 0.000 | 0.997± 0.000 | 0.996± 0.000 | 0.994± 0.001 | 0.990± 0.001 | 0.980± 0.002 | 0.934± 0.009 | 0.328± 0.057 | 0.000± 0.000 |
| **ME**  **(90)** | 1.000±0.000 | 1.000± 0.000 | 1.000± 0.000 | 1.000± 0.000 | 1.000± 0.000 | 1.000± 0.000 | 0.999± 0.000 | 0.999± 0.000 | 0.998± 0.000 | 0.992± 0.001 | 0.728± 0.029 | 0.000± 0.000 |
| **ME**  **(80)** | 1.000±0.000 | 1.000± 0.000 | 1.000± 0.000 | 1.000± 0.000 | 1.000± 0.000 | 1.000± 0.000 | 1.000± 0.000 | 0.999± 0.000 | 0.999± 0.000 | 0.996± 0.001 | 0.814± 0.036 | 0.000± 0.000 |
| **ME**  **(70)** | 1.000±0.000 | 1.000± 0.000 | 1.000± 0.000 | 1.000± 0.000 | 1.000± 0.000 | 1.000± 0.000 | 1.000± 0.000 | 1.000± 0.000 | 0.999± 0.000 | 0.997± 0.000 | 0.865± 0.010 | 0.000± 0.000 |
| **ME**  **(60)** | 1.000±0.000 | 1.000± 0.000 | 1.000± 0.000 | 1.000± 0.000 | 1.000± 0.000 | 1.000± 0.000 | 1.000± 0.000 | 1.000± 0.000 | 0.999± 0.000 | 0.998± 0.000 | 0.900± 0.012 | 0.000± 0.000 |
| **ME**  **(50)** | 1.000±0.000 | 1.000± 0.000 | 1.000± 0.000 | 1.000± 0.000 | 1.000± 0.000 | 1.000± 0.000 | 1.000± 0.000 | 1.000± 0.000 | 1.000± 0.000 | 0.998± 0.000 | 0.913± 0.008 | 0.000± 0.000 |
| **ME**  **(40)** | 1.000±0.000 | 1.000± 0.000 | 1.000± 0.000 | 1.000± 0.000 | 1.000± 0.000 | 1.000± 0.000 | 1.000± 0.000 | 1.000± 0.000 | 1.000± 0.000 | 0.999± 0.000 | 0.926± 0.013 | 0.000± 0.000 |
| **ME**  **(30)** | 1.000±0.000 | 1.000± 0.000 | 1.000± 0.000 | 1.000± 0.000 | 1.000± 0.000 | 1.000± 0.000 | 1.000± 0.000 | 1.000± 0.000 | 1.000± 0.000 | 0.999± 0.000 | 0.931± 0.014 | 0.000± 0.000 |
| **ME**  **(20)** | 1.000±0.000 | 1.000± 0.000 | 1.000± 0.000 | 1.000± 0.000 | 1.000± 0.000 | 1.000± 0.000 | 1.000± 0.000 | 1.000± 0.000 | 1.000± 0.000 | 0.999± 0.000 | 0.936± 0.008 | 0.000± 0.000 |
| **ME**  **(10)** | 1.000±0.000 | 1.000± 0.000 | 1.000± 0.000 | 1.000± 0.000 | 1.000± 0.000 | 1.000± 0.000 | 1.000± 0.000 | 1.000± 0.000 | 1.000± 0.000 | 0.999± 0.000 | 0.943± 0.014 | 0.000± 0.000 |
| **FE** | 1.000±0.000 | 1.000± 0.000 | 1.000± 0.000 | 1.000± 0.000 | 1.000± 0.000 | 1.000± 0.000 | 1.000± 0.000 | 1.000± 0.000 | 1.000± 0.000 | 0.999± 0.000 | 0.952± 0.007 | 0.000± 0.000 |
