# Supplementary material for: Neutral Models of Microbiome Evolution
Source: PLoS Comput Biol. 2015 Jul 22;11(7):e1004365. doi: 10.1371/journal.pcbi.1004365 (PMC4511668; doi:10.1371/journal.pcbi.1004365)
Supplement: S3 Table — (DOCX) [file pcbi.1004365.s003.docx]

**S3 Mean β-diversities ± standard deviations under different combinations of acquisition and environment models**

|  | **EA** | **MA(10)** | **MA(20)** | **MA(30)** | **MA(40)** | **MA(50)** | **MA(60)** | **MA(70)** | **MA(80)** | **MA(90)** | **MA(99)** | **PA** |
| --- | --- | --- | --- | --- | --- | --- | --- | --- | --- | --- | --- | --- |
| **PE** | 0.000±0.000 | 0.000± 0.000 | 0.000± 0.000 | 0.000± 0.000 | 0.000± 0.000 | 0.000± 0.000 | 0.000± 0.000 | 0.000± 0.000 | 0.000± 0.000 | 0.000± 0.000 | 0.000± 0.000 | 0.000± 0.000 |
| **ME**  **(99)** | 0.215±0.001 | 0.216± 0.000 | 0.219± 0.000 | 0.225± 0.000 | 0.234± 0.001 | 0.247± 0.001 | 0.265± 0.001 | 0.293± 0.001 | 0.338± 0.001 | 0.408± 0.007 | 0.253± 0.040 | 0.000± 0.000 |
| **ME**  **(90)** | 0.216±0.001 | 0.217± 0.000 | 0.220± 0.001 | 0.226± 0.001 | 0.234± 0.001 | 0.248± 0.001 | 0.267± 0.001 | 0.296± 0.001 | 0.348± 0.001 | 0.451± 0.002 | 0.581± 0.031 | 0.000± 0.000 |
| **ME**  **(80)** | 0.216±0.001 | 0.217± 0.001 | 0.220± 0.001 | 0.226± 0.001 | 0.235± 0.001 | 0.248± 0.000 | 0.267± 0.001 | 0.297± 0.001 | 0.348± 0.001 | 0.455± 0.002 | 0.671± 0.033 | 0.000± 0.000 |
| **ME**  **(70)** | 0.216±0.000 | 0.217± 0.001 | 0.220± 0.001 | 0.226± 0.000 | 0.235± 0.000 | 0.248± 0.001 | 0.267± 0.001 | 0.297± 0.001 | 0.348± 0.001 | 0.456± 0.002 | 0.720± 0.011 | 0.000± 0.000 |
| **ME**  **(60)** | 0.216±0.001 | 0.217± 0.001 | 0.220± 0.001 | 0.226± 0.001 | 0.235± 0.000 | 0.247± 0.001 | 0.267± 0.001 | 0.297± 0.001 | 0.348± 0.001 | 0.456± 0.002 | 0.756± 0.014 | 0.000± 0.000 |
| **ME**  **(50)** | 0.216±0.000 | 0.217± 0.001 | 0.220± 0.000 | 0.226± 0.000 | 0.235± 0.001 | 0.248± 0.001 | 0.267± 0.001 | 0.297± 0.000 | 0.348± 0.001 | 0.456± 0.002 | 0.769± 0.013 | 0.000± 0.000 |
| **ME**  **(40)** | 0.216±0.001 | 0.217± 0.000 | 0.220± 0.001 | 0.226± 0.000 | 0.235± 0.001 | 0.248± 0.001 | 0.267± 0.000 | 0.297± 0.001 | 0.348± 0.001 | 0.457± 0.002 | 0.782± 0.013 | 0.000± 0.000 |
| **ME**  **(30)** | 0.216±0.000 | 0.217± 0.001 | 0.220± 0.001 | 0.226± 0.001 | 0.235± 0.001 | 0.248± 0.001 | 0.267± 0.001 | 0.297± 0.001 | 0.348± 0.001 | 0.457± 0.002 | 0.784± 0.017 | 0.000± 0.000 |
| **ME**  **(20)** | 0.216±0.000 | 0.217± 0.001 | 0.220± 0.001 | 0.226± 0.001 | 0.235± 0.000 | 0.248± 0.001 | 0.267± 0.001 | 0.298± 0.001 | 0.348± 0.001 | 0.457± 0.001 | 0.791± 0.011 | 0.000± 0.000 |
| **ME**  **(10)** | 0.216±0.001 | 0.217± 0.000 | 0.220± 0.001 | 0.226± 0.001 | 0.235± 0.001 | 0.248± 0.001 | 0.267± 0.001 | 0.297± 0.001 | 0.348± 0.001 | 0.457± 0.002 | 0.795± 0.017 | 0.000± 0.000 |
| **FE** | 0.216±0.001 | 0.217± 0.001 | 0.220± 0.001 | 0.226± 0.001 | 0.234± 0.001 | 0.248± 0.001 | 0.267± 0.001 | 0.297± 0.001 | 0.348± 0.001 | 0.457± 0.002 | 0.805± 0.008 | 0.000± 0.000 |
