# Supplementary material for: Neutral Models of Microbiome Evolution
Source: PLoS Comput Biol. 2015 Jul 22;11(7):e1004365. doi: 10.1371/journal.pcbi.1004365 (PMC4511668; doi:10.1371/journal.pcbi.1004365)
Supplement: S4 Table — (DOCX) [file pcbi.1004365.s004.docx]

**S4 α-diversity under different combinations of acquisition and environment models with log-scaled for MA(X) and ME(Y)**

|  | **EA** | **MA(50)** | **MA(75)** | **MA(87.5)** | **MA(93.8)** | **MA(96.8)** | **MA(98.4)** | **MA(99.2)** | **MA(99.6)** | **MA(99.8)** | **MA(99.9)** | **PA** |
| --- | --- | --- | --- | --- | --- | --- | --- | --- | --- | --- | --- | --- |
| **PE** | 0.000±0.000 | 0.000 ±0.000 | 0.000 ±0.000 | 0.000 ±0.000 | 0.000 ±0.000 | 0.000 ±0.000 | 0.000 ±0.000 | 0.000 ±0.000 | 0.000 ±0.000 | 0.000 ±0.000 | 0.000 ±0.000 | 0.000 ±0.000 |
| **ME**  **(99.9)** | 0.970±0.002 | 0.943 ±0.005 | 0.859 ±0.012 | 0.718 ±0.016 | 0.521 ±0.045 | 0.377 ±0.036 | 0.222 ±0.053 | 0.121 ±0.057 | 0.059 ±0.054 | 0.032 ±0.045 | 0.007 ±0.022 | 0.000 ±0.000 |
| **ME**  **(99.8)** | 0.977±0.001 | 0.959 ±0.003 | 0.907 ±0.005 | 0.785 ±0.011 | 0.596 ±0.023 | 0.427 ±0.033 | 0.262 ±0.057 | 0.124 ±0.072 | 0.063 ±0.083 | 0.022 ±0.041 | 0.001 ±0.003 | 0.000 ±0.000 |
| **ME**  **(99.6)** | 0.981±0.000 | 0.970 ±0.001 | 0.936 ±0.004 | 0.842 ±0.013 | 0.697 ±0.023 | 0.487 ±0.017 | 0.297 ±0.070 | 0.170 ±0.050 | 0.060 ±0.070 | 0.012 ±0.023 | 0.011 ±0.027 | 0.000 ±0.000 |
| **ME**  **(99.2)** | 0.983±0.000 | 0.975 ±0.001 | 0.949 ±0.002 | 0.887 ±0.005 | 0.761 ±0.014 | 0.571 ±0.023 | 0.384 ±0.058 | 0.214 ±0.049 | 0.124 ±0.047 | 0.032 ±0.054 | 0.012 ±0.027 | 0.000 ±0.000 |
| **ME**  **(98.4)** | 0.984±0.000 | 0.977 ±0.000 | 0.958 ±0.001 | 0.913 ±0.004 | 0.820 ±0.008 | 0.664 ±0.019 | 0.480 ±0.033 | 0.281 ±0.081 | 0.157 ±0.067 | 0.071 ±0.056 | 0.031 ±0.051 | 0.000 ±0.000 |
| **ME**  **(96.8)** | 0.984±0.000 | 0.979 ±0.000 | 0.962 ±0.001 | 0.925 ±0.002 | 0.853 ±0.004 | 0.730 ±0.012 | 0.564 ±0.020 | 0.373 ±0.042 | 0.208 ±0.061 | 0.137 ±0.063 | 0.030 ±0.037 | 0.000 ±0.000 |
| **ME**  **(93.8)** | 0.984±0.000 | 0.979 ±0.000 | 0.964 ±0.000 | 0.933 ±0.001 | 0.875 ±0.003 | 0.774 ±0.007 | 0.627 ±0.016 | 0.441 ±0.042 | 0.281 ±0.080 | 0.126 ±0.058 | 0.061 ±0.053 | 0.000 ±0.000 |
| **ME**  **(87.5)** | 0.985±0.000 | 0.980 ±0.000 | 0.965 ±0.000 | 0.937 ±0.001 | 0.884 ±0.002 | 0.799 ±0.003 | 0.680 ±0.009 | 0.531 ±0.021 | 0.353 ±0.062 | 0.219 ±0.069 | 0.095 ±0.048 | 0.000 ±0.000 |
| **ME**  **(75)** | 0.985±0.000 | 0.980 ±0.000 | 0.966 ±0.000 | 0.939 ±0.001 | 0.891 ±0.002 | 0.817 ±0.003 | 0.709 ±0.005 | 0.569 ±0.021 | 0.424 ±0.032 | 0.291 ±0.055 | 0.163 ±0.065 | 0.000 ±0.000 |
| **ME**  **(50)** | 0.985±0.000 | 0.980 ±0.000 | 0.966 ±0.000 | 0.940 ±0.000 | 0.894 ±0.001 | 0.823 ±0.002 | 0.729 ±0.003 | 0.618 ±0.010 | 0.489 ±0.016 | 0.372 ±0.024 | 0.240 ±0.030 | 0.000 ±0.000 |
| **FE** | 0.985±0.000 | 0.980 ±0.000 | 0.966 ±0.000 | 0.940 ±0.001 | 0.895 ±0.001 | 0.828 ±0.002 | 0.739 ±0.003 | 0.634 ±0.007 | 0.523 ±0.011 | 0.401 ±0.031 | 0.289 ±0.053 | 0.000 ±0.000 |
