# Supplementary material for: Neutral Models of Microbiome Evolution
Source: PLoS Comput Biol. 2015 Jul 22;11(7):e1004365. doi: 10.1371/journal.pcbi.1004365 (PMC4511668; doi:10.1371/journal.pcbi.1004365)
Supplement: S5 Table — (DOCX) [file pcbi.1004365.s005.docx]

**S5 γ-diversity under different combinations of acquisition and environment models with log-scales for MA(X) and ME(Y)**

|  | **EA** | **MA(50)** | **MA(75)** | **MA(87.5)** | **MA(93.8)** | **MA(96.8)** | **MA(98.4)** | **MA(99.2)** | **MA(99.6)** | **MA(99.8)** | **MA(99.9)** | **PA** |
| --- | --- | --- | --- | --- | --- | --- | --- | --- | --- | --- | --- | --- |
| **PE** | 0.000±0.000 | 0.000 ±0.000 | 0.000 ±0.000 | 0.000 ±0.000 | 0.000 ±0.000 | 0.000 ±0.000 | 0.000 ±0.000 | 0.000 ±0.000 | 0.000 ±0.000 | 0.000 ±0.000 | 0.000 ±0.000 | 0.000 ±0.000 |
| **ME**  **(99.9)** | 0.986±0.002 | 0.963 ±0.005 | 0.889 ±0.012 | 0.750 ±0.016 | 0.546 ±0.048 | 0.395 ±0.037 | 0.235 ±0.055 | 0.129 ±0.061 | 0.067 ±0.059 | 0.039 ±0.054 | 0.009 ±0.027 | 0.000 ±0.000 |
| **ME**  **(99.8)** | 0.992±0.001 | 0.980 ±0.003 | 0.939 ±0.005 | 0.827 ±0.012 | 0.633 ±0.024 | 0.454 ±0.035 | 0.281 ±0.060 | 0.138 ±0.077 | 0.072 ±0.093 | 0.028 ±0.048 | 0.002 ±0.005 | 0.000 ±0.000 |
| **ME**  **(99.6)** | 0.996±0.000 | 0.990 ±0.001 | 0.969 ±0.004 | 0.892 ±0.014 | 0.749 ±0.026 | 0.525 ±0.020 | 0.325 ±0.072 | 0.190 ±0.055 | 0.067 ±0.076 | 0.016 ±0.028 | 0.015 ±0.034 | 0.000 ±0.000 |
| **ME**  **(99.2)** | 0.998±0.000 | 0.995 ±0.001 | 0.982 ±0.002 | 0.942 ±0.005 | 0.830 ±0.016 | 0.632 ±0.025 | 0.427 ±0.065 | 0.242 ±0.054 | 0.144 ±0.055 | 0.040 ±0.063 | 0.020 ±0.038 | 0.000 ±0.000 |
| **ME**  **(98.4)** | 0.999±0.000 | 0.997 ±0.000 | 0.991 ±0.001 | 0.970 ±0.004 | 0.903 ±0.009 | 0.751 ±0.024 | 0.549 ±0.038 | 0.331 ±0.089 | 0.186 ±0.077 | 0.087 ±0.063 | 0.041 ±0.066 | 0.000 ±0.000 |
| **ME**  **(96.8)** | 1.000±0.000 | 0.999 ±0.000 | 0.995 ±0.001 | 0.984 ±0.002 | 0.945 ±0.004 | 0.844 ±0.014 | 0.666 ±0.025 | 0.450 ±0.052 | 0.256 ±0.068 | 0.174 ±0.077 | 0.040 ±0.043 | 0.000 ±0.000 |
| **ME**  **(93.8)** | 1.000±0.000 | 0.999 ±0.000 | 0.998 ±0.000 | 0.992 ±0.001 | 0.973 ±0.003 | 0.909 ±0.009 | 0.767 ±0.024 | 0.552 ±0.056 | 0.372 ±0.104 | 0.172 ±0.075 | 0.087 ±0.075 | 0.000 ±0.000 |
| **ME**  **(87.5)** | 1.000±0.000 | 1.000 ±0.000 | 0.999 ±0.000 | 0.996 ±0.000 | 0.985 ±0.002 | 0.946 ±0.005 | 0.857 ±0.012 | 0.701 ±0.035 | 0.475 ±0.090 | 0.311 ±0.095 | 0.139 ±0.064 | 0.000 ±0.000 |
| **ME**  **(75)** | 1.000±0.000 | 1.000 ±0.000 | 0.999 ±0.000 | 0.998 ±0.000 | 0.992 ±0.001 | 0.974 ±0.003 | 0.917 ±0.007 | 0.777 ±0.033 | 0.605 ±0.057 | 0.440 ±0.077 | 0.240 ±0.096 | 0.000 ±0.000 |
| **ME**  **(50)** | 1.000±0.000 | 1.000 ±0.000 | 1.000 ±0.000 | 0.999 ±0.000 | 0.996 ±0.000 | 0.985 ±0.002 | 0.952 ±0.006 | 0.882 ±0.016 | 0.742 ±0.032 | 0.589 ±0.036 | 0.379 ±0.029 | 0.000 ±0.000 |
| **FE** | 1.000±0.000 | 1.000 ±0.000 | 1.000 ±0.000 | 0.999 ±0.000 | 0.998 ±0.000 | 0.993 ±0.001 | 0.975 ±0.004 | 0.926 ±0.010 | 0.842 ±0.021 | 0.689 ±0.035 | 0.504 ±0.091 | 0.000 ±0.000 |
