# Supplementary material for: Neutral Models of Microbiome Evolution
Source: PLoS Comput Biol. 2015 Jul 22;11(7):e1004365. doi: 10.1371/journal.pcbi.1004365 (PMC4511668; doi:10.1371/journal.pcbi.1004365)
Supplement: S6 Table — (DOCX) [file pcbi.1004365.s006.docx]

**S6 β-diversity under different combinations of acquisition and environment models with log-scales for MA(X) and ME(Y)**

|  | **EA** | **MA(50)** | **MA(75)** | **MA(87.5)** | **MA(93.8)** | **MA(96.8)** | **MA(98.4)** | **MA(99.2)** | **MA(99.6)** | **MA(99.8)** | **MA(99.9)** | **PA** |
| --- | --- | --- | --- | --- | --- | --- | --- | --- | --- | --- | --- | --- |
| **PE** | 0.000±0.000 | 0.000 ±0.000 | 0.000 ±0.000 | 0.000 ±0.000 | 0.000 ±0.000 | 0.000 ±0.000 | 0.000 ±0.000 | 0.000 ±0.000 | 0.000 ±0.000 | 0.000 ±0.000 | 0.000 ±0.000 | 0.000 ±0.000 |
| **ME**  **(99.9)** | 0.212±0.001 | 0.235 ±0.002 | 0.263 ±0.005 | 0.259 ±0.009 | 0.214 ±0.023 | 0.190 ±0.015 | 0.152 ±0.029 | 0.127 ±0.060 | 0.090 ±0.081 | 0.071 ±0.099 | 0.015 ±0.048 | 0.000 ±0.000 |
| **ME**  **(99.8)** | 0.214±0.000 | 0.241 ±0.001 | 0.289 ±0.003 | 0.302 ±0.007 | 0.266 ±0.012 | 0.227 ±0.019 | 0.188 ±0.036 | 0.137 ±0.073 | 0.086 ±0.111 | 0.048 ±0.083 | 0.003 ±0.008 | 0.000 ±0.000 |
| **ME**  **(99.6)** | 0.215±0.001 | 0.244 ±0.001 | 0.305 ±0.002 | 0.346 ±0.010 | 0.335 ±0.018 | 0.273 ±0.015 | 0.215 ±0.040 | 0.174 ±0.040 | 0.075 ±0.074 | 0.028 ±0.050 | 0.028 ±0.069 | 0.000 ±0.000 |
| **ME**  **(99.2)** | 0.215±0.001 | 0.246 ±0.001 | 0.311 ±0.001 | 0.380 ±0.004 | 0.396 ±0.011 | 0.347 ±0.015 | 0.282 ±0.046 | 0.215 ±0.043 | 0.168 ±0.059 | 0.059 ±0.096 | 0.041 ±0.081 | 0.000 ±0.000 |
| **ME**  **(98.4)** | 0.215±0.000 | 0.247 ±0.001 | 0.314 ±0.002 | 0.399 ±0.004 | 0.456 ±0.008 | 0.435 ±0.021 | 0.368 ±0.027 | 0.283 ±0.059 | 0.194 ±0.096 | 0.119 ±0.081 | 0.073 ±0.115 | 0.000 ±0.000 |
| **ME**  **(96.8)** | 0.216±0.001 | 0.247 ±0.001 | 0.317 ±0.001 | 0.408 ±0.002 | 0.493 ±0.005 | 0.517 ±0.013 | 0.461 ±0.021 | 0.386 ±0.056 | 0.288 ±0.076 | 0.245 ±0.105 | 0.062 ±0.064 | 0.000 ±0.000 |
| **ME**  **(93.8)** | 0.216±0.001 | 0.248 ±0.001 | 0.318 ±0.001 | 0.414 ±0.001 | 0.519 ±0.004 | 0.576 ±0.009 | 0.559 ±0.023 | 0.462 ±0.048 | 0.401 ±0.116 | 0.246 ±0.128 | 0.142 ±0.133 | 0.000 ±0.000 |
| **ME**  **(87.5)** | 0.216±0.000 | 0.248 ±0.001 | 0.318 ±0.000 | 0.418 ±0.001 | 0.530 ±0.002 | 0.616 ±0.005 | 0.644 ±0.013 | 0.599 ±0.044 | 0.479 ±0.096 | 0.390 ±0.109 | 0.203 ±0.095 | 0.000 ±0.000 |
| **ME**  **(75)** | 0.216±0.001 | 0.248 ±0.001 | 0.319 ±0.001 | 0.419 ±0.002 | 0.537 ±0.003 | 0.646 ±0.005 | 0.707 ±0.009 | 0.668 ±0.041 | 0.603 ±0.073 | 0.527 ±0.091 | 0.326 ±0.133 | 0.000 ±0.000 |
| **ME**  **(50)** | 0.216±0.001 | 0.248 ±0.001 | 0.319 ±0.001 | 0.419 ±0.002 | 0.540 ±0.002 | 0.660 ±0.005 | 0.743 ±0.007 | 0.772 ±0.019 | 0.727 ±0.034 | 0.663 ±0.056 | 0.487 ±0.044 | 0.000 ±0.000 |
| **FE** | 0.216±0.001 | 0.247 ±0.001 | 0.319 ±0.001 | 0.420 ±0.002 | 0.542 ±0.002 | 0.666 ±0.004 | 0.765 ±0.004 | 0.814 ±0.016 | 0.818 ±0.024 | 0.761 ±0.032 | 0.618 ±0.112 | 0.000 ±0.000 |
