# Supplementary figures and images for: Neutral Models of Microbiome Evolution
Source: PLoS Comput Biol. 2015 Jul 22;11(7):e1004365. doi: 10.1371/journal.pcbi.1004365 (PMC4511668; doi:10.1371/journal.pcbi.1004365)

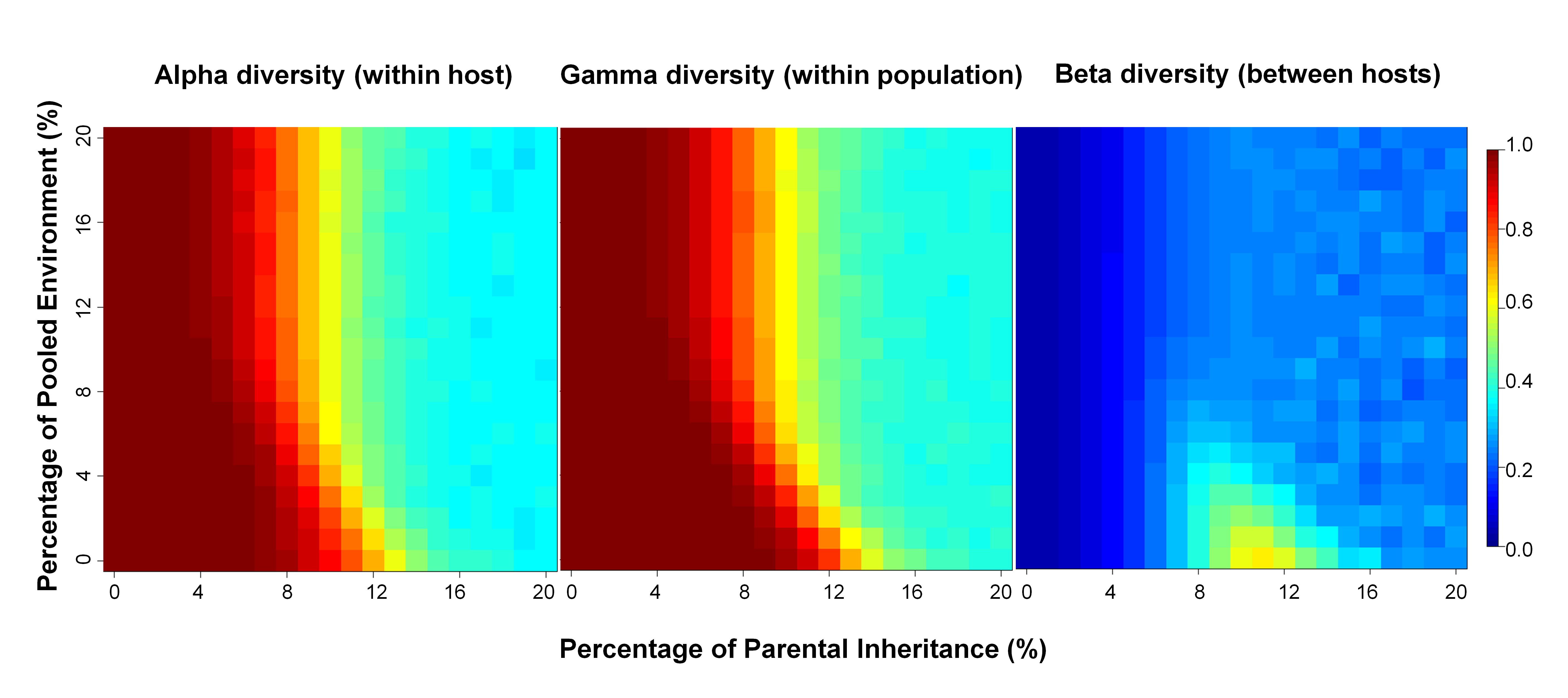

Supplement: S1 Fig — The diversity patterns are also represented with heatmaps in a similar way (the proportion of parental inheritance = 1–0.5x with x ranging from 0 to 20; the proportion of pooled environmental component = 1–0.5y with y ranging from 0 to 20), and a similar pattern were still observed regardless of the increasing numbers of hosts, microbes and microbial taxa. (TIF) [file pcbi.1004365.s008.tif]

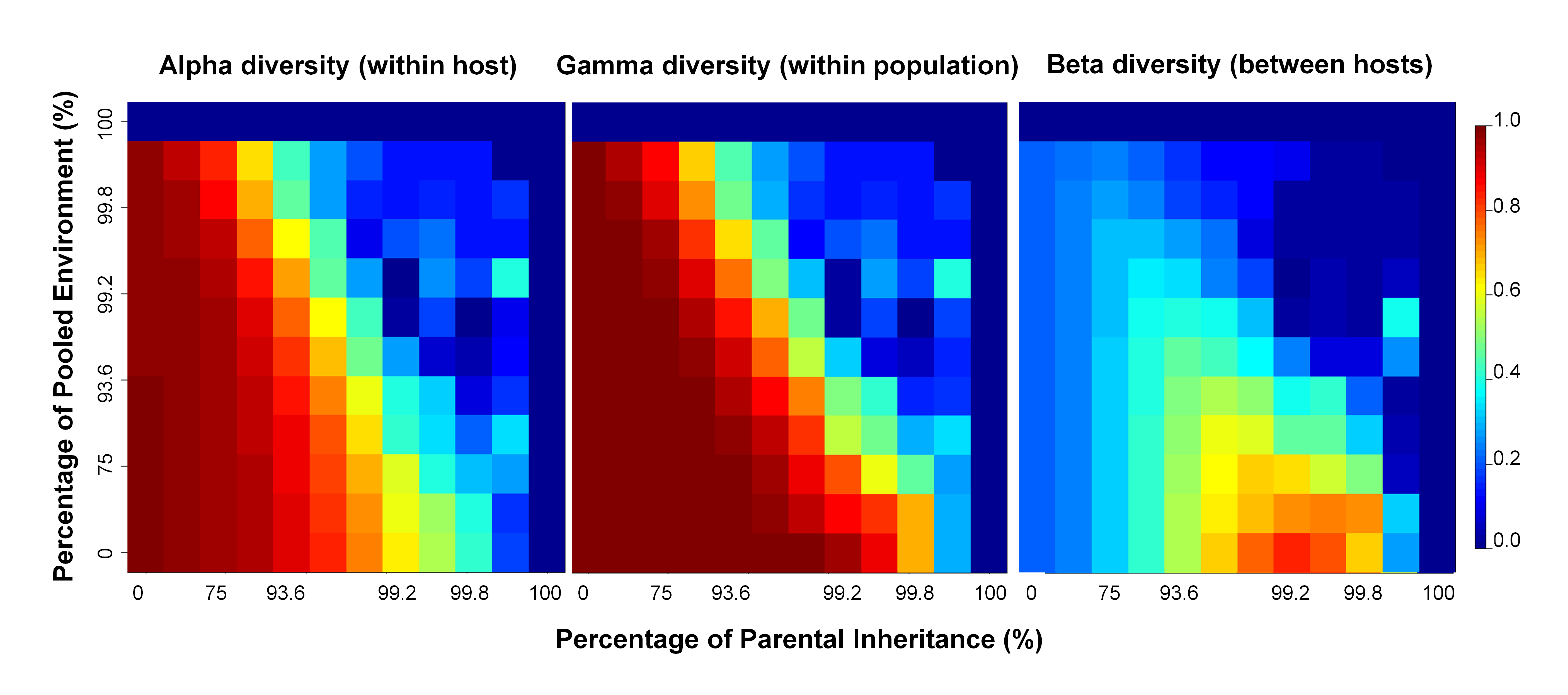

Supplement: S2 Fig — The same log-scales as in Fig 4 is used, and each square represents a re-estimated diversity value from a fitted Dirichlet-Multinomial distribution with ten replicates. (TIF) [file pcbi.1004365.s009.tif]
